# Italian Quacks and Lymph

**Published:** 1891-04

**Authors:** 


### Italian Quacks and the Lymph.

The *Basler Nachrichteu's* special Italian correspondent, writing from Milan, states that Koch's discovery is there degraded in a highly disgraceful manner. Circulars are sent around by obscure doctors announcing that they have just received a sufficient of Koch's Lymph with which they are prepared to cure consumptive patients. The lymph is in no case genuine, and yet phthisical patients, of whom there are many in Milan, flock to fill the coffers of these medical *chevaliers d'industrie.* The authorities have, as yet, taken no steps to prevent or punish this abuse. The following is a translation of the inscription on the sign-board of a "dispensary:" "Teeth extracted, corns cut, and tuberculosis cured after Professor Koch's method."—*British Med. Journal.*

Treatment of Dysentery by Irrigation of Lower Bowels. Dr. Koritin reports fifteen cases of dysentery cured by irrigation of the lower bowels. He had nine cases of the diphtheritic form of dysentery and six of the catarrhal (according to Virchow's division of the disease). In two diphtheritic cases a solution of carbolic acid, 1 drachm to six pounds of water was used; and in seven cases, grains xx to six pounds of water. In the catarrhal form: in two cases, grains xx; in one, grain x to six pounds of water, and in three other cases pure water was used. The author, after fully describing each case, concludes his interesting article, saying that though he has used besides the irrigation some of the popular internal and external remedies, nevertheless, he thinks that the course of the disease, as given in his description, was modified by the irrigation.

Medical Achievement in China. It is said of Dr. Kerr, a medical missionary in Canton, that he has in the past thirty-six years, treated over 520,000 patients, and has prepared twenty-seven medical and surgical books. He has trained one hundred medical assistants, chiefly Chinese. China now possesses one hundred and four hospitals and dispensaries, at which, in 1889, more than 348,000 patients received treatment. *Med. News.*

Absorption of Tumors by Electricity.—Dr. Moritz Meyer reports a number of instructive cases in which he caused the disappearance of tumors by percutaneous galvanization and faradication. In 1859 he removed, by means of the faradic current, an indurated tumor of the neck the size of a man's head, which the celebrated Langenbeck had regarded as inoperable. Several years ago he produced, in this way, absorption of a mass of callus of the size of a fist, on the elbow joint, and last year obtained the same result in a case of callus formation on the second phalanx of the right index finger which had rendered the patient unable to work. After thirty-seven sittings the tumor had disappeared, and the function of the joint was completely restored. In a case of chronic synovitis, which was only temporarily improved by massage, galvanization effected a perfect cure, and this was also observed in a case of gouty deposits in the sheaths of the tendons. For galvanization the author employs flexible electrodes of various size adapting themselves to the surface of the body.—*Berlin. Klin. Wochenschr.*, November 30, 1890.—*Int. Journal of Surgery.*

Florida's University.—The plans of the buildings for the projected university at Tarpon Springs, Fla., of which Dr. Charles E. Sajous, of Philadelphia is president, are now in hand, and it is stated lectures are to be commenced on October of next year. At first there will be only one building, but others are to be added. The lines of Harvard are to be closely followed, and there will be faculties of medicine, law, theology, arts, veterinary surgery, and dentistry.

The university has been mainly founded for students of debilitated health who are compelled by the severity of the Northern winters to forego study during those months and to travel in the South. The medical department will give special attention to the study of climatic treatment of pulmonary affections. There will be a sanitarium and colony founded in connection with the university, and the whole will form a health resort for the winter months corresponding to the European Riviera.

PHOSPHATE OF LIME.—Phosphate of lime is a medicine much under-valued. It builds up the constitution by aiding digestion and nutrition, and enables the bony system to grow much faster than without its use. It can be made into a syrup and given to children with rachitis, or it may be administerd in combination with the hypophosphites and other well-known preparations, as manufacture l by reliable pharmaceutical houses.

During pregnancy the lactophosphate of lime should be given for the growth of the fœtus, especially in women of such constitution that the drain on the system is very great, and even then the child will be born sickly and with weak bones.

MICROBES IN THE STOMACH.—Dr. Kianovski details in the last two numbers of the *Vrach* some observations he has recently made upon the bacteria contained in the stomach before and during digestion, with the object of determining the effect of the gastric juice upon them. He found that the fasting stomach of a healthy person always contained a large number of microbes, and that in the earlier part of digestion the number of these bodies is also considerable, and that it depends mainly on the number introduced by the food, saliva, etc. Notwithstanding this, the gastric juice, or rather, perhaps the free hydrochloric acid in it, tends to exert a decidedly destructive influence upon the microbes. No effect appears to be produced upon the process of digestion by these bodies.—*The Lancet.*

KOCH'S CURE FOR CONSUMPTION.—It will be remembered that Professor Koch created a certain sensation at the International Medical Congress by stating that his unceasing efforts to find a remedy for tuberculosis seemed, at last, about to be crowned with success. Since then experiments with the new remedy have been going on in Professor Senator's department of the Charite. The time is, of course, much too short for any thing like definite results, but as the experiments proceed the veil of secrecy will no doubt be lifted.—Berlin Correspondence *British Medical Journal.*

INFECTIOUS ORIGIN OF TETANUS.—The view that tetanus is (*Bulletin de la Gazette Medicale*) infectious has long been prevalent in France and other countries. Recently Leyden has reported to the Society for Internal Medicine of Berlin, three cases of tetanus in man, one of which was cured. In the three cases the bacillus of Nicolaier was found in the neighborhood of the "tetanic" wound; inoculations successively upon the mouse, rabbit, and dog, producing the characteristic disease in each instance. With the first two animals the result was fatal. The interesting conclusions in these cases relate to pathogenesis and treatment. We can believe with Leyden that tetanus is a virulent disease, the symptoms being caused by the contact of the poison with the nervous centres, without the intervention of anatomical lesions that are problematical. For the same reason we can admit that the chances of a cure augment as the disease lengthens, and if the patient survive the sixth day there is a good chance of recovery. The indications for treatment point more strongly to local antisepsis, than in any other microbic or virulent disease.

PYOCTANINE. The aniline colors have among them several members of the fraternity of antiseptic substances. Pyoctanine is the name of the violet and yellow members. Other aniline colors will probably assert their properties in that line within a short time. Heretofore we could often find out to what antiseptic substances a surgeon was partial by the various odors that permeated him as with a garment, but at present the hues that variegate his hands will give him away. Members of the profession that are endowed with an artistic eye will be able to dress wounds with picturesque effect. Two liberal an employment of primary colors will be described as rather harsh treatment. There will be simple, steady, reliable elderly gentlemen who will be content with a monochromatic dressing, while others, often of lesser years and more æsthetic tendencies, will stake their reputation and their patient's welfare upon an application of substances cunningly mingled, so as to resemble more in general effect the every-day patchwork quilt of our aunts and the coat of Joseph. *International Jour. of Surgery.*

